# Improving Substance Use Disorder Treatment Training in Medical School

**DOI:** 10.7759/cureus.54638

**Published:** 2024-02-21

**Authors:** Moronkeji Fagbemi

**Affiliations:** 1 Internal Medicine, BronxCare Health System, New York, USA

**Keywords:** addiction risk stratification, brief interventions (bi), substance use screening, pain treatment, medical school curriculum, behavioral change intervention, addiction stigmatization, medical education and training, medical education, substance use disorder (sud)

## Abstract

Substance use disorder (SUD) remains a major cause of morbidity and mortality in the United States and globally. Even though a lot of proposals have been implemented to help combat the opioid epidemic and are to be applauded, there remain a lot of changes that need to be made at the level of medical school training of physicians. It will take a paradigm shift to effect a lasting change in the culture around SUD treatment.

This will include a review of the curriculum, which is still skewed towards the management of established diseases rather than prevention and screening, the changing of the lingo of stigmatization of patients and the disease, which in turn affects treatment utilization. These changes should also emphasize risk stratification, the ready application of the United States Preventive Services Task Force screening recommendations for drug and alcohol screening, and the use of recommended drinking limits for men and women readily in patient evaluation, coupled with prompt intervention. There should be a concerted effort to build skills in proven evidenced-based behavioral therapy complementary to existing effective pharmacological therapies.

The examinations by medical schools and the medical examining bodies should reflect these changes. Despite all our efforts in the treatment of established SUD so far, we are not going to treat our way out of the “drug epidemic” without emphasis on prevention and intervention, especially at the grassroots of medical education.

## Editorial

Substance use disorder (SUD) remains a significant cause of morbidity and mortality, with 109,680 drug overdose deaths in 2022 and overdose deaths more than tripled over the last decade [1]. It is estimated that well over 46.3 million people (16.5 percent of the population) aged 12 or older fulfill the Diagnostic and Statistical Manual of Mental Disorders, Fifth Edition (DSM-5) criteria for SUD, and more than 27 million people in the United States reported that they are using illicit drugs or misusing prescription drugs, with nearly a quarter of adults and adolescents reporting binge drinking in the past month [2]. The massive economic burden and annual economic impact of substance misuse are estimated to be $249 billion for alcohol misuse and $193 billion for illicit drug use [2].

A lot of proposals have been implemented to help combat the opioid epidemic, including the development of prescription monitoring programs in states, the Center for Disease Control and Prevention (CDC) education of limiting initial opioid use for acute pain to 7 days or less, the periodic mandated pain management training by opioid prescribers, and lately the removal of the requirement that healthcare providers possess a DATA-Waiver, commonly referred to as an X-Waiver, to prescribe buprenorphine to treat opioid use disorder (OUD). While these are to be applauded, more changes must be made from the onset of physician training at the medical school level. How did we get here? It started with the over-prescription of opioid pain medications, and now the number of opioid overdoses has mushroomed in recent years due to increased utilization of opioids in the management of pain, especially chronic pain, but also due to the increasing use of highly potent and addictive opioids appearing on the illicit drug market. It will take a paradigm shift from the grassroots of medical education to effect a lasting change, and this is where medical school education comes into play. After all, these are the future leaders going to be saddled with developing effective answers to this growing battle with SUD.

A review of the curriculum of most medical schools shows an attempt at the integration of basic, clinical, social, and health systems sciences throughout the continuum of medical education, but addiction education is still skewed towards the management of established diseases rather than prevention and screening. Furthermore, most of the clinical knowledge and skill set about SUD for many students are during their brief psychiatry core rotation sandwiched among the myriad of competing topics, with only a few students electing to use some of their electives towards gaining further knowledge in SUD evaluation, assessment, and treatment [3]. In his piece titled “Most Doctors Are Ill-Equipped to Deal with the Opioid Epidemic. Few Medical School Teach Addiction,” written in the New York Times on September 10, 2018, investigative journalist Jan Hoffman alluded that while most medical schools now offer some education about opioids, only 15 of the 180 American programs teach addiction as including Alcohol, tobacco, and other drugs and the content varies widely ranging from one lecture to few weeks usually during the student’s psychiatry rotation.

While it is essential to master the basic facts of addictive disorders, including pathogenesis, diagnostic points, and major treatments, it is even more important to help students understand that addiction is a treatable disease (like other chronic relapsing diseases) and not a “moral failure”. Many physicians and medical students are not aware that there are effective treatments for SUD and that the patients are not a “lost cause”. It is mind-boggling that less than 25% of individuals with OUD are enrolled in evidence-based opiate agonist therapy (OAT) [4].

Medical School education should be a crucial time to adjust the lingo of would-be clinicians. First, the patients are individuals with SUD and not “addicts or alcoholic”. If there is one thing we have learned from the opioid crisis, it is that it traverses every age, race, gender, and socioeconomic class. Feeling stigmatized can make people with SUD less willing to seek treatment, while negative stereotypes about people with SUD can make others (including clinicians and other allied workers) feel pity, fear, and even anger toward patients [5].

Secondly, the new terminology is substance use disorder, which could be mild, moderate, or severe based on the Diagnostic and Statistical Manual of Mental Disorders, 5th Edition (DSM-5) criteria, not “Drug habit”. The use of the word “abuse” instead of the word used when dealing with illicit substances or misuse when dealing with prescription medication was found to have a high association with negative judgments and punishment.

There must be a concerted effort to focus education and training on students knowing and applying the United States Preventive Services Task Force (USPSTF) recommendations for drug and Alcohol screening, the recommended drinking limits for men and women in patient evaluation so that emphasis can be placed on prevention, patient education to maintain abstinence, and non-problem use, as well as timely intervention for a patient with at-risk use. Medical school training must emphasize the importance of prescribing opioids appropriately, including evaluating risk stratification and assessment for addiction. There must be an emphasis on the stepwise approach to the use of analgesics and a multimodal approach to pain treatment, including the use of non-pharmacological therapies. Furthermore, the students should be trained to recognize behavioral changes in patients that may signal the development of Substance Use Disorder, including doctor shopping.

It would be beneficial for medical schools to incorporate the learning of evidenced-based behavioral and interventional skills such as brief intervention, motivational interviewing, and cognitive behavioral therapy centered around SUD treatment.

Brief interventions with its six common elements summarized by the acronym FRAMES, consisting of Feedback, Responsibility, Advise, Menu for change, Empathy, and enhancing Self-efficacy, when combined with referral to appropriate treatment services, have shown to be efficacious in reducing substance use and its harmful consequences [6]. In helping medical students build their motivational interviewing skills, they develop a different perspective in which you, as a clinician, adopt a different style from solving problems for people to encouraging them to solve them for themselves. Teaching and training in the basics of cognitive behavioral therapy (CBT) allow students to help patients address problematic thoughts and feelings required to overcome addiction. It also has the added benefit of helping treat co-occurring psychiatric disorders prevalent in many of the patients [7]. This will improve the likelihood of physicians referring patients for further CBT sessions with trained and licensed psychologists, counselors, and psychiatrists.

Finally, the above areas of emphasis should be reflected in the National Board of Medical Examiners (NBME) shelf examination and the United States Medical Licensure Examinations (USMLE).

In conclusion, despite all our efforts in the treatment of established addictive disorders, we are not going to treat our way out of the “drug epidemic” without serious emphasis on prevention and intervention at the grassroots of medical education. This will require more than a slight tweaking of the curriculum, but a commitment to integrating the neuroscience of addiction at every level of medical school training, changing our dismissive culture and stigmatization of patients while modeling compassionate care with evidence-based treatments, coupled with the continuous pain management and opioid prescription training, as well as the development of useful behavioral interventional skills in medical students. The earlier we begin, the better our chance of success.
